# Comparison of airway pressure release ventilation (APRV) versus biphasic positive airway pressure (BIPAP) ventilation in COVID-19 associated ARDS using transpulmonary pressure monitoring

**DOI:** 10.1186/s12871-025-02904-7

**Published:** 2025-02-01

**Authors:** Sandra Emily Stoll, Tobias Leupold, Hendrik Drinhaus, Fabian Dusse, Bernd W. Böttiger, Alexander Mathes

**Affiliations:** 1 Department of Anesthesiology and Intensive Care Medicine, Faculty of Medicine and University Hospital Cologne, University of Cologne, Cologne, Germany; 2 Department of Anesthesiology, Montefiore Medical Center, Albert Einstein College of Medicine, New York, USA; 3 Department of Anesthesiology and Intensive Care Medicine (Study Center), Faculty of Medicine, University of Cologne, University Hospital Cologne, Kerpener Str. 62, 50937 Cologne, Germany

**Keywords:** Transpulmonary pressure, COVID, ARDS, SARS CoV-2, CARDS, Atelectrauma, VILI, Ventilator associated lung injury, APRV, BIPAP

## Abstract

**Background:**

APRV has been used for ARDS in the past. Little is known about the risk of ventilator- induced lung- injury (VILI) in APRV vs. BIPAP in the management of in COVID19-associated ARDS (CARDS). This study aimed to compare transpulmonary pressures (TPP) in APRV vs. BIPAP in CARDS in regard to lung protective ventilator settings.

**Methods:**

This retrospective, monocentric cohort study (ethical approval: 21-1553) assessed all adult ICU- patients with CARDS who were ventilated with BIPAP vs. APRV and monitored with TPP from 03/2020 to 10/2021. Ventilator-settings / -pressures, TPP, hemodynamic and arterial blood gas parameters were compared in both modes.

**Results:**

20 non- spontaneously breathing patients could be included in the study: Median TPPendexpiratory was lower / negative in APRV (-1.20mbar; IQR − 4.88 / +4.53) vs. positive in BIPAP (+ 3.4mbar; IQR + 1.95 / +8.57; *p* < .01). Median TPPendinspiratory did not differ. In APRV, mean tidal- volume per body- weight (7.05 ± 1.28 vs. 5.03 ± 0.77 ml; *p* < .01) and mean airway- pressure (27.08 ± 1.67 vs. 22.68 ± 2.62mbar; *p* < .01) were higher. There was no difference in PEEP, peak-, plateau- or driving- pressure, compliance, oxygenation and CO_2_- removal between both modes.

**Conclusion:**

Despite higher tidal- volumes / airway-pressures in APRV vs. BIPAP, TPPendinspirator*y* was not increased. However, in APRV median TPPendexpiratory was negative indicating an elevated risk of occult atelectasis in APRV- mode in CARDS. Therefore, TPP- monitoring could be a useful tool for monitoring a safe application of APRV- mode in CARDS.

**Supplementary Information:**

The online version contains supplementary material available at 10.1186/s12871-025-02904-7.

## Introduction

During the pandemic of SARS-CoV-2, many Intensive Care patients presented with Coronavirus Disease 19 (COVID 19)-associated-ARDS (CARDS). Though fulfilling the Berlin definition, CARDS differs from ARDS of other pathophysiology. Patients suffering from CARDS often show signs of severe hypoxia requiring mechanical ventilation [1, 2]. Mortality of CARDS remains high and ventilator-associated lung injury (VILI) might be a major contributor.

Airway pressure release ventilation (APRV) has been used as a “rescue mode” of ventilation in managing patients with ARDS of other origin in the past to improve oxygenation and to keep the lung open (open lung concept) more consistently and more homogeneously [3]. APRV and other modes of biphasic positive airway pressure (BIPAP) represent a pressure targeted intermittent mandatory ventilation ranging biphasically between an upper and lower pressure level. But in contrast to BIPAP, APRV delivers continuous positive pressure (P_high_) for a preset of prolonged inspiratory time (T_high_) with intermittent releases to a lower pressure level (P_low_) at a preset of a shortened expiratory time (T_low_). This shortened expiratory time in APRV is supposed to create an intrinsic PEEP which ideally avoids cyclic derecruitment and reduces cyclical opening and collapse of lungs. But unfortunately, in APRV intrinsic PEEP and driving pressure can be difficult to measure and may be unreliable predictors of atelectasis.

Moreover, as prolonged inspiratory times resulting in increased mean airway pressures, are characteristic for APRV, this mode is supposed to establish alveolar recruitment, improvement of lung compliance and optimization of ventilation in relation to alveolar perfusion [4]. Vice versa, other studies highlight the potential risk of baro- or volutrauma in APRV [5]. This might especially be problematic in CARDS, a typically heterogenous lung disease.

Unfortunately, conventional ventilator monitoring is an insufficient detector for the risk of VILI. Transpulmonary pressure (TPP) could be a useful tool to detect an increased risk for baro-, volu- and atelectrauma in mechanically ventilated patients. So far, there is little knowledge about the risk of VILI in APRV vs. BIPAP in CARDS [1, 6, 7]. Therefore, this study aimed to assess whether there are differences regarding the risk of volu-, baro- and atelectrauma in patients suffering from CARDS comparing ventilation in APRV- vs. BIPAP- mode by monitoring endinspiratory and endexpiratory TPP.

## Materials and methods

### Setting

After ethical review board approval (21-1553-retro), this observational single center study retrospectively analyzed patients admitted to a surgical German 14-bed ICU between March 2020 to October 2021. The study hospital was a quaternal, referral and teaching hospital with approximately 3500 admissions to Intensive Care Medicine per year. During the COVID pandemic one 14-bed subunit of the ICU focused on the treatment of patients with respiratory failure due to CARDS.

### Study design

We retrospectively screened 139 ventilated COVID-19 subjects for the fulfillment of the following inclusion criteria: All adult (≥ 18 years) SARS-CoV-2 positive subjects suffering from CARDS (ARDS as per Berlin classification), who were admitted to the Intensive Care Unit between March 2020 and October 2021, who were ventilated in BIPAP- mode initially and who consecutively had a trial of APRV for refractory hypoxemia were included in this study. Furthermore, all subjects involved in this study were monitored with TPP as per clinical standard for ARDS patients and as per clinical decision of the treating physician. APRV was chosen in all subjects as a “rescue mode” of ventilation to improve oxygenation in refractory hypoxemia by decision of the treating clinician. Patients without ARDS not requiring intubation, patients who did not have a trial of ventilation in BIPAP- and APRV- mode and patients without TPP monitoring were excluded from the study.

### Ventilator settings

All subjects were ventilated with Hamilton C6 ventilators (Hamilton Medical AG, Bonaduz Swiss) which have an auxiliary port for monitoring esophageal and transpulmonary pressure via a separate pressure line. Initial ventilator mode after intubation for all subjects was BIPAP- mode (DUOPAP, Hamilton Medical AG, Bonaduz Swiss) as per hospital standard. BIPAP was used as a pressure regulated mode allowing asynchronous spontaneous breathing but without pressure assist. Initial ventilator settings were chosen as per ARDSnetwork recommendations for lung protective ventilation as per hospital protocol: Tidal volume of ≤ 6 ml / kg predicted body weight (PBW), Plateau pressure ≤ 30mbar, Driving pressure ≤ 16mbar, PEEP as defined by ARDSnet table and permissive hypercapnia (if pH > 7.2) [8]. In case of transfer to APRV to improve oxygenation, this mode was set according to initial BIPAP- settings with P_high_ <30mbar (aiming for the same plateau pressure as in BIPAP / DUOPAP- mode), P_low_ ≤5mbar and the T_high_ at 5 s. As per hospital protocol T_low_ was initiated at 0.5 s. Then the expiratory flow waveform was assessed and T_low_ adjusted accordingly to achieve expiratory flow termination at 75% of peak expiratory flow. This setting of T_low_ avoids emptying of the lung, creating an endexpiratory pressure (extrinsic PEEP plus intrinsic PEEP) counteracting lung derecruitment. In both ventilation modes pressure support ventilation (PSV) was removed as per hospital standard to avoid hyperinflation on the upper pressure level by additional spontaneous breaths. In both BIPAP and APRV automated tube compensation was applied in all patients. All subjects with CARDS were deeply sedated (Glasgow Coma scale Score (GCS) 3 and Richmond Agitation and Sedation Scale Score (RASS) -5)) or even paralyzed to avoid spontaneous ventilation.

### Transpulmonary pressure monitoring

TPP- monitoring was initiated in subjects suffering from CARDS as per decision of the treating physician to optimize ventilator settings (plateau pressure, PEEP, tidal volume): In case of an endexpiratory TPP < 0mbar PEEP was increased (targeting an endexpiratory TPP of 0-10mbar) whereas in case of an endinspiratory TPP > 20-25mbar plateau pressure or tidal volume was reduced (as per hospital protocol) to target an endinspiratory TPP < 20mbar. TPP was always measured before and after a change of the ventilation mode or ventilator settings, in case of patient deterioration and as per the treating physician´s request. TPP was monitored using esophageal pressure as a surrogate for pleural pressure with a special nasogastric tube (NGT) (Nutrivent™, Löwenstein Medical) with an inflatable esophageal balloon. This NGT was positioned like a regular nasogastric tube after exclusion of contraindications. TPP was consecutively calculated by the difference of plateau pressure in inspiratory hold (representing alveolar pressure) and esophageal pressure. For the description of the correct positioning of the NGT Nutrivent™ tube and the explanation of the derived parameters (endinspiratory TPP, endexpoiratory TPP, elastance- derived TPP) please see the summary in the supplement (text 1).

### Comparison of APRV- and BIPAP- mode

All subjects included in this study were ventilated in BIPAP in supine position initially with settings according to the ARDSnet recommendations [8]. Patients with refractory hypoxemia in BIPAP- mode had a trial in APRV in supine position at the discretion of the intensive care consultant, according to the local practice. In this analysis, esophageal pressure, TPP, ventilator settings and pressures, arterial blood gas (ABG)-results (PaO_2_, PaCO_2_), sedation requirements, grade of sedation (GCS- and RASS- Score) and hemodynamic parameters (heart rate, systolic, mean and diastolic blood pressure, dosage of vasopressor support) were assessed in BIPAP and APRV. To increase the monitored timeframe in both ventilator modes, the mean / median values of the three last measurements of these parameters in BIPAP were compared to the respective values of the first three measurements in APRV. Intervals between the timepoints of the individual measurements were 1–2 h apart.

### Data management and collection

Demographic data, illness severity scores (Sequential Organ failure assessment Score (SOFA) and Acute Physiology and Chronic Health Evaluation (APACHE III)- Score), variant of SARS-CoV-2, comorbidities, comedications, results from biochemistry and ABGs were retrieved from the patients’ electronic and paper chart. Furthermore, data of hemodynamic parameters, ventilator settings and measurements as well as measurements of esophageal pressure and TPP were collected. Data was retrieved by two independent examiners.

### Ethical approval

This study was approved by the Ethics Committee of the University of Cologne, Germany (21-1553-retro) as a low risk with a waiver of individual patient informed consent due to the retrospective character and the analysis of pseudonymized data. Informed consent was not taken from the individual patient. The study was conducted according to the declaration of Helsinki.

### Sample size

Since this study was a retrospective study, it was only possible to assess a convenience sample of all the subjects fulfilling the inclusion criteria.

### Statistical analysis

Continuous variables were compared using means with standard deviation (SD) and medians with interquartile ranges (IQR) according to data type and distribution. Categorical variables were compared using frequency counts and percentages. For comparisons of all parameters in APRV- vs. BIPAP paired-samples Student’s t-test or Wilcoxon signed-rank test as appropriate for continuous variables and chi-square test with Yates correction for categorical variables was used. A two-sided p-value ≤ 0.05 was chosen to indicate statistical significance. Analyses were performed using SPSS Statistics version 27 (IBM, Armonk, NY, USA).

## Results

A total of twenty subjects with CARDS could be included in this study. Of these twenty subjects, 13 (65%) subjects were male. The mean age of the subjects was 60.59 ± 13.04 years, mean BMI was 33.63 ± 7.77 kg/m^2^, mean APACHE Score was 21.50 ± 10.69 and mean SOFA-score was 7.65 ± 3.63. Most subjects (18/20; 90%) presented with the alpha -variant / B1.1.7 of SARS CoV-2. Demographic data are given in Table 1 in the supplement.

### Neurological parameters

Due to the severity of CARDS, all subjects included in this trial were sedated in APRV and BIPAP- mode to a Richmond Agitation and Sedation Scale (RASS)- Score of -5 and presented with a Glasgow Coma Score (GCS) of 3 to avoid spontaneous breathing. Four subjects (20%) included in this study were paralyzed to establish lung protective ventilation (see Table 2 in the supplement).

### Hemodynamic parameters

There were neither differences regarding mean heart rate, mean systolic or mean diastolic blood pressure nor regarding vasopressor- (noradrenaline-) requirements in APRV vs. BIPAP. All hemodynamic variables are given in Table 3 in the supplement.

### ABG-results

There were no differences regarding mean FiO_2_, mean SpO_2_, mean PaO_2_, mean PaCO_2_, mean pH, mean base excess, mean lactate, mean estimated shunt (calculated by the ABG machine) and mean PaO_2_/FiO_2_ ratio comparing APRV vs. BIPAP (see Table 4 in the supplement).

### Ventilator parameters

All subjects included in this study were ventilated in BIPAP-mode initially. The mean ventilation time in BIPAP- mode was 478.58 ± 334.99 vs. 5.4 ± 1.12 h in APRV- mode.

Given the severity of CARDS spontaneous breathing was suppressed by sedation in all study subjects. The mean number of days before a trial of APRV was initiated was 3.05 ± 2.31 days after ICU admission as per decision of the treating physician and also terminated by the treating physician e.g. in case of hemodynamic instability or failure to improve gas exchange with this “rescue mode”. All patients transferred to APRV- mode suffered from ARDS as per Berlin classification (PaO2/ FiO2 < 300mmHg). In APRV- mode, mean tidal volume per predicted body weight, mean airway pressure and intrinsic PEEP were significantly higher compared to BIPAP. Vice versa, mean respiratory rate was significantly lower in APRV than in BIPAP as was mean set (extrinsic) PEEP. There was no difference in mean total PEEP (intrinsic PEEP plus extrinsic PEEP), peak-, plateau or driving- pressure (calculated by the difference of plateau pressure minus extrinsic PEEP and intrinsic PEEP) in APRV vs. BIPAP. Also, no difference could be found regarding the mechanical power, the compliance or resistance of the patients’ lungs in comparing both modes. All ventilator parameters are given in Table 5 in the supplement.

### Transpulmonary pressure

**Fig. 1:**
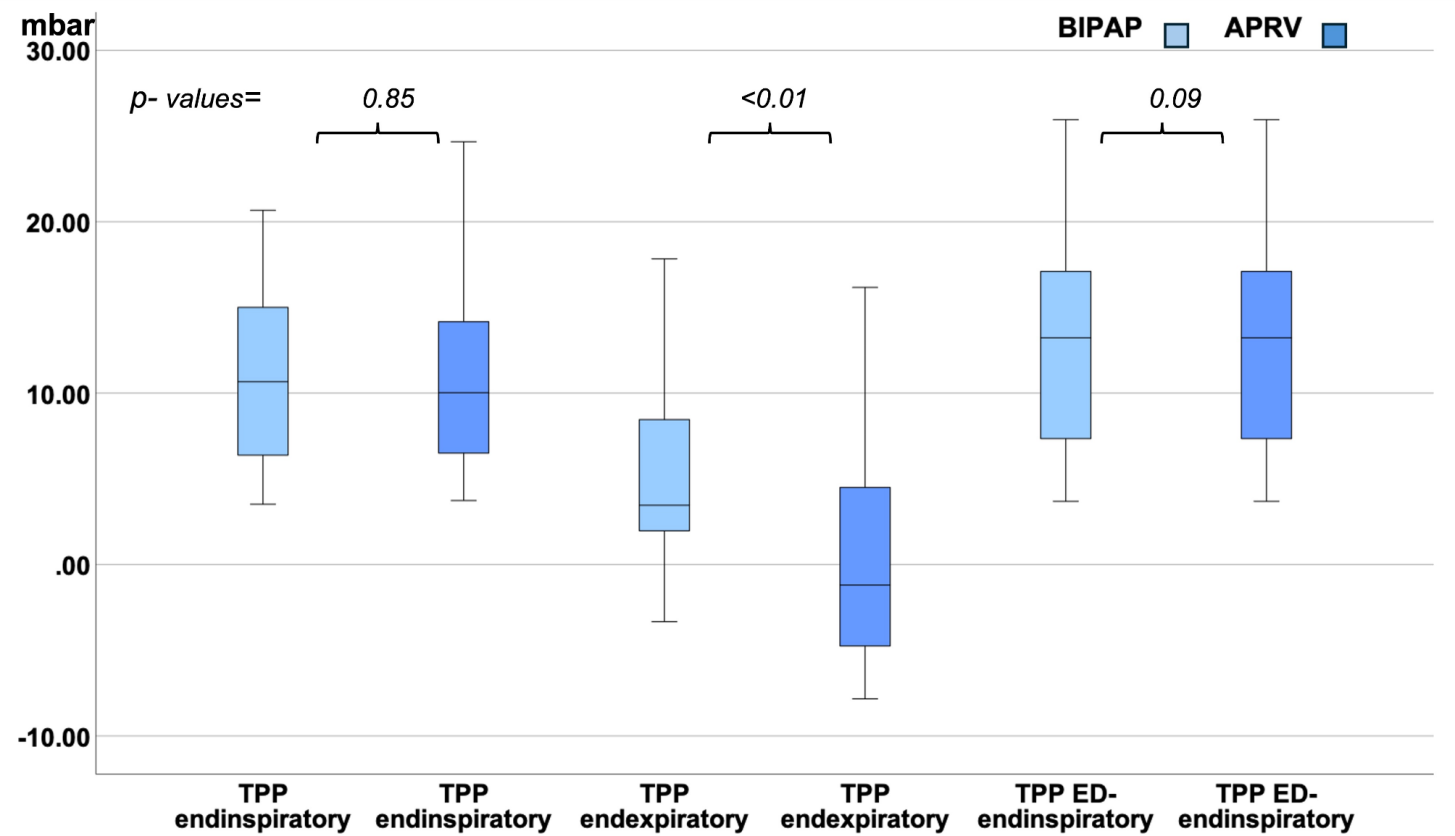
Median endinspiratory, Median endexpiratory and median elastance derived (ED) endinspiratory transpulmonary pressure (TPP) in all included study subjects (in mbar) in APRV vs. BIPAP

**Fig. 2:**
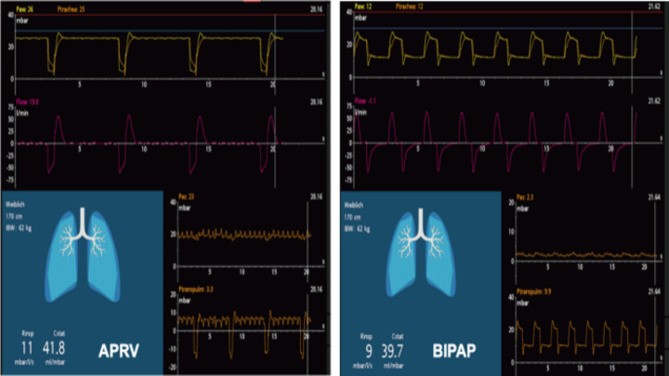
TPP-monitoring in APRV vs. BIPAP (same patient) without spontaneous breathing. The negative endexpiratory TPP In APRV vs. positive in BIPAP indicates an increased risk of atelectasis in APRV- mode

As measured transpulmonary pressures were not normally distributed, we compared medians and Interquartile range (IQR) of TPP in APRV vs. BIPAP: There was a significant difference in median endexpiratory TPP in APRV vs. BIPAP. In contrast to BIPAP, in APRV-mode median endexpiratory TPP was lower and negative (-1.2mbar; IQR − 4.88/4.53) vs. positive (3.45mbar; IQR 1.95 / 8.57; *p* < .01) in BIPAP (see Figs. 1 and 2; Table 5 in the supplement). Furthermore, median transpulmonary driving pressure was significantly higher in APRV (*p* < .01; see Table 5 in the supplement) vs. BIPAP. In contrast, mean PEEP, plateau-, peak, and driving pressure did not differ in both modes (see Table 5 in the supplement). Moreover, there was no difference in median endinspiratory TPP (10.02mbar; IQR 6.5/14.25 in APRV vs. 10.67mbar; IQR 6.08 / 15.5; *p* = .85) and median elastance-derived endinspiratory TPP (ED-TPP endinspiratory) in APRV vs. BIPAP (*p* = .09 see Fig. 1 / Table 5 in the supplement). Median endinspiratory TPP was < 20mbar in both modes.

**Fig. 3:**
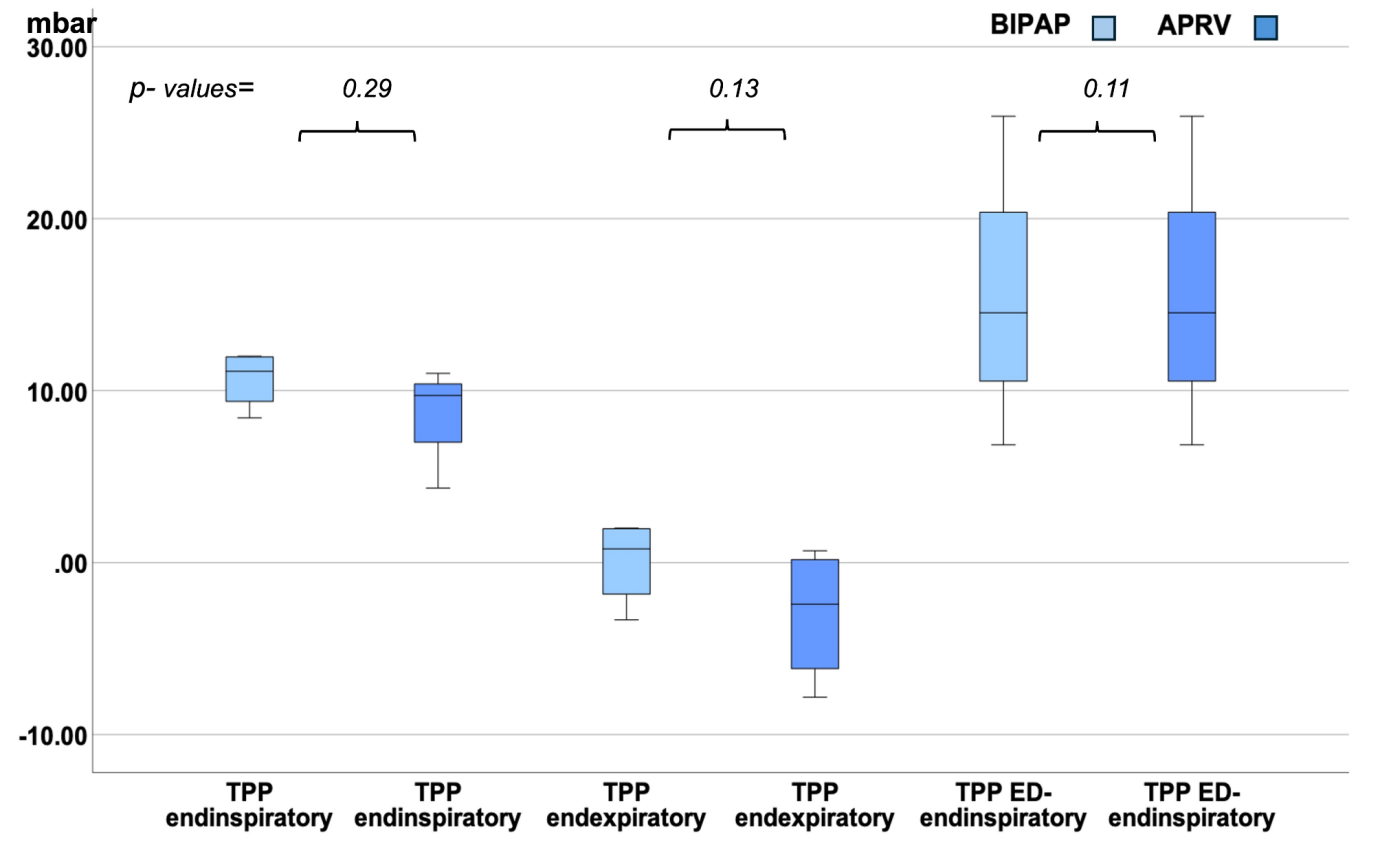
Median endinspiratory, Median endexpiratory and median elastance-derived endinspiratory TPP (ED-TPP) in mbar in APRV vs. BIPAP in paralyzed subjects

**Fig. 4:**
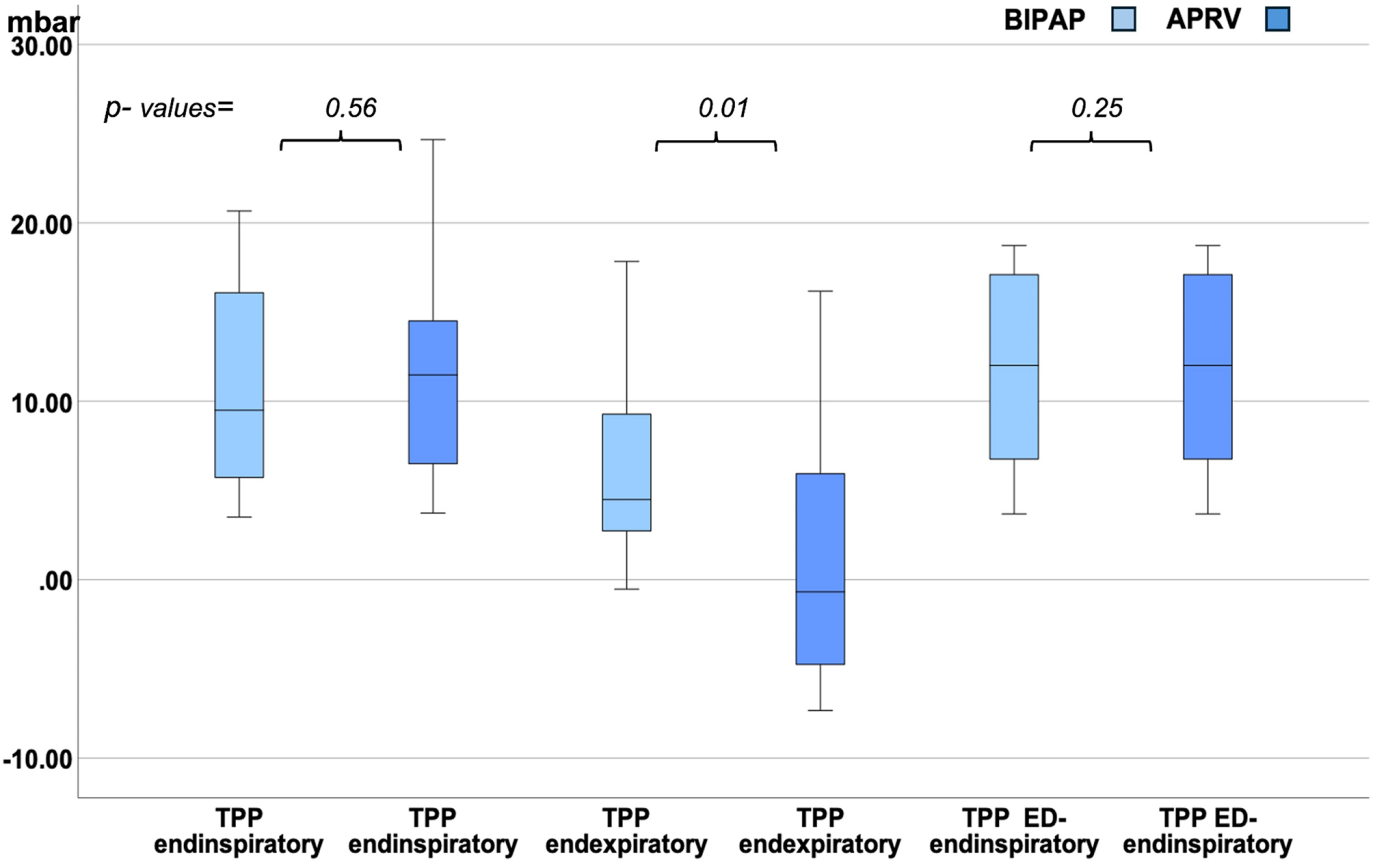
Median endinspiratory, Median endexpiratory and median elastance-derived endinspiratory TPP (ED-TPP) in mbar in APRV vs. BIPAP in non-paralyzed subjects

Only 4 out of 20 patients were paralyzed. Therefore, we additionally analyzed TPP in the different subgroups of non- paralyzed patients and paralyzed patients (see Figs. 3 and 4).

In each of both groups (non- paralyzed and paralyzed subjects) median endexpiratory TPP was also lower and negative in APRV vs. positive in BIPAP. Median endinspiratory TPP and elastance derived endinspiratory TPP was < 20mbar in both modes without significant differences (see Figs. 3 and 4).

### Posthoc power analysis of comparing endexpiratory TPP in APRV vs. BIPAP

In a posthoc power analysis, using t-test for comparison of dependent means (endexpiratory TPP in APRV vs. BIPAP) with an effect size of 0.69 (Cohen´s d), an alpha-error of 0.05, a sample size of 20 achieved a power of 0.93.

## Discussion

### Key findings

In our study, mean tidal volumes and mean plateau pressures presented to be higher in APRV vs. BIPAP. Nevertheless, neither in APRV nor in BIPAP, an increased endinspiratory TPP nor a difference in mechanical power could be detected. In contrast, endexpiratory TPP was negative in APRV vs. positive in BIPAP indicating an increased risk of atelectasis in APRV which would not have been detected without TPP monitoring.

Relation to previous studies.

Multiple studies already defined lung protective ventilation for volume- assist control mode of ventilation and ventilator settings in BIPAP- mode can be adjusted to these recommendations [8]. But these recommendations are not applicable for APRV- mode. Lung protective settings in APRV have been described using the time- controlled adaptive ventilation (TCAV) method. In APRV, lung protective ventilation should not aim for a specific intrinsic PEEP or target certain tidal volumes. Instead, the goal is to minimize dynamic alveolar strain by adjusting ventilation to the individual lung´s mechanical characteristics [9]. Unfortunately, so far, there is no clear definitions for APRV- settings and this circumstance impedes direct comparison of different studies. There is limited evidence of animal or human studies to support the titration of tidal volumes per kg of predicted body weight in APRV and lung protective ventilation in APRV should not target specific tidal volumes per predicted body weight [5, 10, 11]. Despite the lack of clear recommendations regarding the setting of APRV- mode, some previous studies claimed a benefit of using APRV for recruitment of alveoli in cases of ARDS [12–14], where conventional modes of mechanical ventilation failed to improve gas exchange [1]. Several animal studies could show an improvement of oxygenation in APRV by increasing ventilation in the dependent areas of the lung [5, 15–19]. Regarding human studies analyzing ARDS patients, APRV showed to improve PaO2/FiO2 ratio and was able to reduce FiO2 requirements [20, 21]. A meta- analysis by Carsetti et al. was able to reveal a reduction of ventilator- free days at day 28 in APRV- mode compared to conventional ventilator modes [14]. Regarding the impact of APRV- mode on mortality of ARDS patients there is inconsistent evidence so far. Whereas Carsetti et al. [14] and a study by Zhou et al. [20] were able to show that APRV- mode was associated with a reduced mortality, most studies in the past only detected an improvement of oxygenation without any mortality benefit of APRV- mode [9, 22–25]. On the contrary, an Australian prospective, randomized, single center study by Zorbas et al. 2021 could even show a higher 90-day mortality and fewer ventilator-free days independent of the patients‘ severity of illness in APRV versus low tidal volume BIPAP [6].

In our study, we could not see any improvement in oxygenation and CO_2_- removal or shunt reduction as confirmed by other previous studies [31]. The lack of improvement of oxygenation in APRV in our study might be due to the fact that the patients included in our study were mostly treated in APRV- mode for only ≤ 6 h.

Nevertheless, APRV has also been seen critically in the past for the potential to promote VILI. This might be especially the case in spontaneous breathing on the upper pressure level resulting in an increased transpulmonary pressure with an increased risk of volu- and barotrauma. In our study, APRV presented with higher tidal volumes and mean airway pressures compared to BIPAP but this did not result in harmfully increased endinspiratory TPP in APRV as confirmed by other studies in the past [26, 27]. In contrast to increased lung volumes at endinspiration, increased lung volumes at endexpiration in APRV compared to BIPAP are theoretically assumed to promote a reduction of dynamic alveolar strain and to prevent atelectrauma and VILI [5, 28]. Nevertheless, APRV carries a risk of occult atelectrauma especially with an increased T_low_ (which is not adjusted to achieve expiratory flow termination at 75% of peak expiratory flow) [5, 7, 29, 30]. A negative endexpiratory transpulmonary pressure in APRV vs. BIPAP which may be associated with atelectasis was revealed in our study as well. These signs were not predictable by assessing plateau pressure, total PEEP (set extrinsic PEEP plus intrinsic PEEP) or driving pressure on the ventilator screen. In APRV, intrinsic PEEP and driving pressure can be difficult to measure and are unreliable predictors of atelectasis as seen in findings from compute modelling in the past [5]. Conclusively, in our study signs of an increased risk of atelectasis were revealed in TPP monitoring only. In our study Tlow was set at ≤ 0.5 s to achieve expiratory flow termination at 75% of peak expiratory flow as per recommendations of the TCAV approach [10, 20, 31]. Despite the adjustment of ventilator settings in APRV according to the results of the TPP monitoring negative endexpiratory TPP values could not be eliminated by the treating team.

### Limitations and strengths

Our study has several limitations. The retrospective, monocentric setting of our study restricted to a small number of patients limits the generalizability of the data. Additionally, all patients were ventilated in BIPAP first and consecutively in APRV- mode (on 3.05 ± 2.31 days after ICU admission) as a rescue- mode for refractory hypoxemia. This leaves the time effect as a potential confounder and the data can probably not be extrapolated to APRV- mode as a primary mode of ventilation. But as shown in our study, patients were not deteriorating regarding oxygenation, decarboxylation, lung compliance, lung resistance or hemodynamic stability during the study interval assessed. Therefore TPP- measurements during the examined interval in BIPAP and APRV- mode should not be affected by a time component.

Another limitation of our study is, that TPP was not monitored continuously but only at certain timepoints and that only 4 patients of our study were paralyzed. TPP values may not be comparable in non-paralyzed patients and paralyzed patients and TPP values are more prone to artifacts in spontaneous breathing. But all patients in this study were sedated to a RASS Score of -5 and a GCS Score of 3 to avoid spontaneous breathing. None of the patients included in our study were breathing spontaneously. Moreover, TPP as derived from esophageal pressure monitoring, does not reflect all lung regions equally but mainly represents esophageal pressure midesophageal between non-dependent and dependent lung areas. Same can be stated about the intrinsic PEEP which can vary in different regions of the lungs as CARDS is a heterogenous lung disease [5]. To overcome this problem, endinspiratory TPP values were measured directly with the plateau- derived method and additionally calculated using the elastance-derived method [32]. Another limitation of our study is that the duration of ventilation in APRV was potentially too short to see a benefit regarding oxygenation and decarboxylation. In our study, APRV was terminated in most cases within ≤ 6 h as an increased risk of atelectrauma was witnessed in form of a negative endexpiratory TPP, despite adjustment of ventilator settings as the reduction of T_low_. Since the same patients were ventilated in BIPAP and APRV consecutively, we did not assess outcome parameters such as length of invasive ventilation, length of ICU- / Hospital- stay and 30-day mortality in our study.

On the other hand, our study also has certain strengths. To the best of our knowledge, this is the first analysis comparing APRV vs. BIPAP using TPP monitoring in CARDS patients in a clinical setting. Further, as our intensive care unit was focused on the treatment of SARS-CoV-2 patients at the above-mentioned time-period, therapy was highly standardized within a well-educated team. Thus, despite being a retrospective study, the level of standardization may be assumed to be extremely homogenous.

### Implications

Regarding implications, APRV seems to have many theory-based advantages in ARDS, but evidence of large, randomized multicenter trials comparing APRV to other modes of ventilation for CARDS patients are lacking so far [5]. APRV is highly dependent on adjusting ventilator settings individually to the patients’ needs. Inadequate ventilator settings carry a high risk of unwitnessed derecruitment or volu- and barotrauma of the lung leading to VILI [9].

## Conclusion

Despite of higher mean tidal volumes and higher mean airway pressures in APRV vs. BIPAP, both modes did not present an increased risk of volu- or barotrauma regarding median endinspiratory TPP. In contrast, in APRV- mode, median endexpiratory TPP was negative (versus positive in BIPAP) implying an increased risk of atelectasis in APRV which would have remained unwitnessed without TPP-monitoring. Conclusively, TPP monitoring could be a useful tool for a safer application of APRV- mode in patients suffering from CARDS.

## Electronic supplementary material

Below is the link to the electronic supplementary material.

Supplementary Material 1 [33, 34]

## Data Availability

The data that support the findings of this study are not openly available due to reasons of sensitivity and are available from the corresponding author upon reasonable request. Data are located in controlled access data storage at University Hospital Cologne, Germany.

## Abbreviations

ABG: Arterial blood gas analysis
APACHE: Acute physiology and chronic health evaluation
APRV: Airway pressure release ventilation
ARDS: Acute respiratory distress syndrome
BIPAP: Biphasic positive airway pressure
CARDS: COVID 19- related ARDS
COVID 19: Coronavirus Disease 19
FiO_2_: Inspired oxygen fraction
ICU: Intensive care unit
IQR: Interquartile range
iNO: Inhaled nitric oxide
IPPV: Intermittent positive pressure ventilation
IQR: Interquartile range (25.-75. Percentile)
LTV: Low tidal volume
NGT: Nasogastric tube
P_aw_: Airway pressure
P_es_: Esophageal pressure
P_high_: Higher pressure level
P_low_: Pressure on the lower pressure level
PaO_2_: Partial pressure of oxygen
PEEP: Positive end-expiratory pressure
PSV: Pressure support ventilation
SARS CoV-2: Severe acute respiratory syndrome Coronavirus 2
SD: Standard deviation
SOFA: Sepsis related organ failure assessment
T_high_: Time on the higher-pressure level
T_low_: Time on the lower-pressure level
TPP: Transpulmonary pressure
VILI: Ventilator associated lung injury
VS: Versus
