# Supplementary material for: Comparison of airway pressure release ventilation (APRV) versus biphasic positive airway pressure (BIPAP) ventilation in COVID-19 associated ARDS using transpulmonary pressure monitoring
Source: BMC Anesthesiol. 2025 Feb 1;25:52. doi: 10.1186/s12871-025-02904-7 (PMC11786409; doi:10.1186/s12871-025-02904-7)
Supplement: Supplementary file 1 — Supplementary Material 1 [33, 34] [file 12871_2025_2904_MOESM1_ESM.docx]

**Comparison of Airway pressure release ventilation (APRV) versus biphasic positive airway pressure (BIPAP) ventilation in COVID-19 associated ARDS using transpulmonary pressure monitoring.**

**Supplement Content: page nr**

**Text 1: Summary of the correct positioning of the NGT Nutrivent^TM.^ and 1/2**

**description of the different derived TPP- parameters**

**Table 1: Demographic parameters of subjects included in the study 2/3**

**Table 2: Neurological parameters in APRV vs BIPAP with no 4**

**differences between the groups.**

**Table 3: Mean hemodynamic parameters of 3 measurements in APRV 4**

**vs 3 measurements in BIPAP with no differences between the**

**groups.**

**Table 4: Mean ABG results of 3 measurements in BIPAP- vs 3 4**

**measurements in APRV-mode with no significant differences.**

**Table 5: Mean Ventilator parameters in APRV vs BIPAP and median**

**transpulmonary pressure parameters. 4/5**

**Text 1:**

**Summary of the correct positioning of the NGT Nutrivent^TM.^ and description of the different derived TPP- parameters**

The correct position of the NGT Nutrivent^TM^ tube was checked by the method recommended by Baydur et al.^[33]^ in an endinspiratory hold maneuver and by a positive occlusion test with external manual pressure in an endexpiratory hold maneuver^[34]^ by assessing the ratio of the changes of esophageal pressure (ΔP_es_) and the changes of airway pressure (ΔP_aw_). Moreover, the correct position was confirmed by cardiac oscillations in the TPP curve. Correct position of the nasogastric tube was checked before each measurement of TPP as per hospital standard. Given the fact that in supine position there is a pressure gradient of pleural pressure increasing from non-dependent to dependent lung areas, esophageal pressure mainly represents midesophageal regions of the lung between the sternum and the vertebra.^[32]^ Therefore, endexpiratory TPP for assessment of signs of atelectasis in the dependent lung was detected in an expiratory hold maneuver. Endinspiratory TPP >20-25mbar indicating signs of hyperinflation (risk of baro- or volutrauma) in the non-dependent lung was firstly assessed in an endinspiratory hold maneuver (direct endinspiratory TPP). But previous data has shown that direct measurements of endinspiratory TPP underestimate the risk of hyperinflation especially in the non-dependent lung regions, which are at highest risk of hyperinflation.^[32]^ Therefore, to surrogate a more precise value of endinspiratory TPP in sedated, non-spontaneous breathing patients, endinspiratory TPP was additionally calculated by using the elastance-derived-method:^[32]^

*Endinspiratory TPP =*

*Plateau pressure x (Elastance of the lung/ Elastance of the respiratory system).*

For this study, we therefore assessed both endinspiratory TPPs: The plateau-pressure based direct endinspiratory TPP and the elastance derived endinspiratory TPP.

**Table 1: Demographic parameters of subjects included in the study**

| **Parameter** | **N=20** | |
| --- | --- | --- |
|  | **Mean ±SD** | **Median (IQR)** |
| **Age (years)** | 60.59±13.04 | 63.62 (57.25-70.5) |
| **BMI (kg/m^2^)** | 33.63±7.77 | 33.33 (26.26-38.37) |
|  | **(n / %)** | |
| **Sex (male)** | 13/20 (65%) | |
| **COVID variant**  *Wild COVID variant*  *United Kingdom (alpha; B1.1.7) COVID variant*  *South African (beta; B.1.351) COVID variant* | 1/20 (5%)  18/20 (90%)  1/20 (5%) | |
| **Past medical history**  *-Diabetes mellitus*  *-Insulin dependent diabetes*  *-Obesity*  *-Arterial hypertension*  *-Cardiovascular disease*  *-Cardiac Dysrhythmia*  *-Renal insufficiency*  *-Renal replacement therapy*  *-Pulmonary*  *-COPD*  *-Smoking*  *-Alcoholism*  *-Organ Transplant*  *-Thrombosis*  **Pre-Hospital Medication**  *-ACE blocker*  *-NSAID*  *-Statins*  *-Corticosteroids* | 7/20 (35%)  2/20 (10%)  14/20 (70%)  14/20 (79%)  4/20 (20%)  2/20 (10%)  3/20 (15%)  1/20 (5%)  5/20 (25%)  3/20 (15%)  2/20 (10%)  1/20 (5%)  1/20 (5%)  2/20 (10%)  4/20 (20%)  5/20 (25%)  6/20 (30%)  3/20 (15.8%) | |
| **Proning**  **Inhaled nitric oxide**  **Renal replacement therapy**  **Tracheostomy** | 18/20 (90%)  11/20 (55%)  5/20 (25%)  14/20 (70%) | |
| **30-day mortality** | **(n / %)**  12/20 (60%) | |
| **Variables on admission to ICU Mean ±SD Median (IQR)** | | |
| FiO_2_ | 0.78±0.17 | 0.8 (0.62-0.9) |
| SpO_2_  (%) | 91.36±3.41 | 91.5 (89.43-93.75) |
| PaO_2_ (mmHg) | 70.63±16.04 | 67.00 (58.10-81.35) |
| pH | 7.31±0.13 | 7.31 (7.17-7.43) |
| PCO_2_ (mmHg) | 58.24±28.47 | 46.50 (37.25-77.68) |
| PaO_2_/FiO_2_ ratio (mmHg) | 100.48±38.26 | 84.00 (73.00-133.50) |
| Highest Shunt (%) | 33.42±7.99 | 32.90 (27.75-37.45) |
| SOFA Score | 7.65±3.63 | 7 (4.00-10.00) |
| APACHE II Score | 21.50±10.68 | 20.50 (12.00-28.00) |
| Lactate (mmol/l) | 1.85±0.95 | 1.52 (1.10-2.80) |
| White cell count (10^9^/l) | 11.01±6.26 | 9.90 (5.54-15.33) |
| Neutrophile count (10^9^/l) | 13.91±21.29 | 7.44 (4.43-13.91) |
| Lymphocytes (10^9^/l) | 0.86±0.60 | 0.65 (0.40-1.31) |
| Neutrophil / lymphocyte ratio | 21.89±32.75 | 10.08 (5.62-18.06) |
| CRP (mg/l) | 128.11±68.53 | 123.1 (74.6-171.55) |
| Procalcitonin (ug/l) | 0.39±0.32 | 0.3 (0.2-0.5) |
| Interleucin-6 (ng/l) | 81.55±92.89 | 42.00 (23-82) |
| Ferritin (ug/l) | 1841.43±1432.22 | 1421.50 (1067.5-2560.75) |
| Troponin (ug/l) | 0.04±0.04 | 0.03 (0.01-0.09) |
| *APACHE II Score= Acute Physiology And Chronic Health Evaluation II- Score, ACE- blocker= Angiotensin converting enzyme blocker, BMI= Body mass index, BPAP= Biphasic positive airway pressure, COPD= Chronic obstructive Disease, COVID= Coronavirus Disease, CRP= C reactive protein, FiO_2_= fraction of oxygen, ICU= Intensive Care Unit, IQR= Interquartile range, n= number, NSAID= Nonsteroidal paCO_2_= arterial carbon dioxide pressure, paO_2_= arterial partial pressure, SD= Standard deviation, SOFA= Sepsis-related organ failure assessment-Score, , SpO_2_= Saturation of oxygen.* | | |

**Table 2: Neurological parameters in APRV vs BIPAP with no differences between the groups.**

| **Parameter** | **APRV (N=20)** | **BIPAP (N=20)** | ***P*-value** |
| --- | --- | --- | --- |
|  | **Mean± SD / Median (IQR)** | |  |
| *RASS-Score* | -5±0 / -5 (-5/-5) | -5±0 / -5 (-5/-5) | - |
| *GCS-Score* | 3±0 / 3 (3/3) | 3±0 / 3 (3/3) | - |
|  | **N (%)** | |  |
| *Muscle Relaxation* | 4/20 (20%) | 4/20 (20%) | - |
| *APRV= Airway pressure release ventilation, BPAP= biphasic positive airway pressure, GCS= Glasgow Coma Scale, IQR= Interquartile range, n= number, RASS Score= Richmond- Agitation-Sedation-Scale- Score, SD= standard deviation* | | | |

**Table 3: Mean hemodynamic parameters of 3 measurements in APRV vs 3 measurements in BIPAP with no differences between the groups.**

| **Parameter** | **APRV (N=20)** | **BIPAP (N=20)** | **P-value** |
| --- | --- | --- | --- |
|  | **N (%)** | |  |
| *Heart rhythm* | Sinus rhythm 20/20 (100%) | Sinus rhythm 20/20 (100%) | - |
|  | **Mean± SD** | |  |
| *Mean heart rate / min* | 81.38±19.53 | 81.55±15.19 | .98 |
| *Mean Systolic blood pressure (mmHg)* | 116.75±16.27 | 123.03±15.86 | .22 |
| *Mean diastolic blood pressure (mmHg)* | 58.55±8.14 | 58.48±9.61 | .98 |
| *Mean mean blood pressure (mmHg)* | 76.73±9.93 | 79.48±9.02 | .37 |
| *Mean dose of noradrenaline μg/kg/min* | 0.1±0.10 | 0.10±0.10 | .84 |

**Table 4: Mean ABG results of 3 measurements in BIPAP- vs 3 measurements APRV-mode with no significant differences.**

| **Parameter** | **APRV (N=20)** | **BIPAP (N=20)** | ***P*-value** |
| --- | --- | --- | --- |
|  | **Mean ± SD** | |  |
| *Mean FiO_2_* | 0.51±0.20 | 0.56±0.21 | .46 |
| *Mean SpO_2_ (%)* | 95.77±1.93 | 95.83±2.07 | .92 |
| *Mean PaO_2_ (mmHg)* | 98.58±21.17 | 98.05±25.03 | .94 |
| *Mean PCO_2_ (mmHg)* | 51.22±11.62 | 53.19±12.90 | .62 |
| *Mean pH* | 7.35±0.05 | 7.35±0.06 | .61 |
| *Mean base excess (mmol/l)* | 2.47±5.26 | 2.76±4.94 | .86 |
| *Mean Lactate (mmol/l)* | 1.68±0.46 | 1.61±0.42 | .59 |
| *Mean estimated shunt (%)*  *calculated by ABG machine* | 22.08±12.95 | 20.17±7.40 | .57 |
| *Mean PaO_2_/FiO_2_ ratio (mmHg)* | 211.08±75.17 | 195.66±72.06 | .51 |

**Table 5: Mean Ventilator parameters and median TPP parameters in APRV vs BIPAP.**

| **Ventilator Parameter** | **APRV (N=20)** | **BIPAP (N=20)** | ***P*-value** |
| --- | --- | --- | --- |
|  | **Mean ± SD** | |  |
| *Mean TV / PBW (ml)* | 7.05±1.28 | 5.03±0.77 | <0.01 |
| *Mean set, extrinsic PEEP (mbar)*  *on the ventilator* | 4.58±1.31 | 17.25±3.07 | <0.01 |
| *Mean intrinsic PEEP (mbar)*  *on the ventilator* | 15.69±4.68 | 0.71±0.74 | <0.01 |
| *Mean total PEEP (mbar)*  *on the ventilator* | 20.28±4.61 | 17.96±3.12 | 0.62 |
| *Mean Respiratory rate / min* | 15.97±5.34 | 26.55±2.14 | <0.01 |
| *Mean Peak-pressure (mbar)* | 30.55±2.26 | 29.05±2.89 | 0.08 |
| *Mean Thigh (sec)* | 5.00±0.00 | 1.00±0.03 | <0.01 |
| *Mean Tlow (sec)* | 0.51±0.02 | - | - |
| *Mean mean airway pressure (mbar)* | 27.08±1.67 | 22.68±2.62 | <0.01 |
| *Mean plateau pressure (mbar)* | 28.90±2.22 | 26.85±3.44 | 0.31 |
| Mean driving pressure (mbar) | 8.70±3.52 | 8.79±3.20 | 0.94 |
| Mean compliance (ml/mbar)  calculated by the ventilator | 48.23±14.66 | 47.17±19.39 | 0.85 |
| Mean resistance (mbar/l/sec)  calculated by the ventilator | 10.33±3.91 | 9.82±2.20 | 0.61 |
| Mean mechanical power (J/min)  Calculated by the formula  MP= 0.098*RR*TV*(PEEP+ 0.5 (Pplat-PEEP) + (Ppeak-Pplat)) | 20.73±9.07 | 23.02±3.97 | 0.31 |
| Mean ventilation time (hours) | 5.4±1.12 | 478.58±334.99 | <0.01 |
| **TPP- Parameter** | **APRV (N=20)** | **BIPAP (N=20)** | ***P*-value** |
|  | **Median (IQR)** | |  |
| Median endexpiratory transpulmonary pressure (mbar) | -1.20  (-4.88 / 4.53) | 3.45  (1.95 / 8.57) | 0.04 |
| Median endexpiratory esophageal pressure (mbar) | 24.20  815.67 / 28.85) | 15.52  (9.74 / 19.73) | 0.01 |
| Median endinspiratory transpulmonary pressure (in mbar) in direct plateau- derived measurement | 10.02  (6.5 / 14.25) | 10.67  (6.08 / 15.50) | 0.91 |
| Median endinspiratory transpulmonary pressure (ED-TTP; mbar) in indirect elastance- derived measurement | 15.07  (13.34 / 18.11) | 13.23  (7.09 / 17.29) | 0.10 |
| Median endinspiratory esophageal pressure (mbar) | 18.33  (15.09 / 22.08) | 16.75  (11.75 / 19.58) | 0.35 |
| Median transpulmonary driving pressure (mbar) | 9.22  (7.13 / 11.64) | 5.75  (3.43 / 8.23) | 0.01 |
| *APRV= Airway pressure release ventilation, BPAP= biphasic positive airway pressure, mbar= millibar, n= number, MP= Mechanical Power in J/min, PBW= predicted body weight in kg, PEEP= Positive end- expiratory pressure in mbar, Ppeak= Peak pressure in mbar, Pplat= Plateau pressure in mbar, RR= respiratory rate per minute, SD= Standard deviation, sec= second, Thigh= Time on upper pressure level, Tlow= time on lower pressure level, TPP= Transpulmonary pressure, TV= tidal volume* | | | |
